# Dose-Dependent Induction of Differential Seizure Phenotypes by Pilocarpine in Rats: Considerations for Translational Potential

**DOI:** 10.3390/medicina60101579

**Published:** 2024-09-26

**Authors:** Dolika Vasović, Olivera Stanojlović, Dragan Hrnčić, Nikola Šutulović, Milena Vesković, Aleksandar J. Ristić, Nebojša Radunović, Dušan Mladenović

**Affiliations:** 1 Clinical Centre of Serbia, University Eye Hospital, Pasterova 2, 11000 Belgrade, Serbia; dolika.vasovic@gmail.com; 2 Institute of Medical Physiology “Richard Burian”, Faculty of Medicine, University of Belgrade, Višegradska 26/II, 11000 Belgrade, Serbia; 3 Institute of Pathophysiology “Ljubodrag Buba Mihailović”, Faculty of Medicine, University of Belgrade, Dr Subotića 9, 11000 Belgrade, Serbia; 4 Neurology Clinic, Clinical Center of Serbia, Dr Subotića 6, 11000 Belgrade, Serbia; 5 Serbian Academy of Sciences and Arts, Kneza Mihaila 35, 11000 Belgrade, Serbia

**Keywords:** pilocarpine, seizures, behavioral changes, EEG, rats

## Abstract

*Background and Objectives*: Pilocarpine is used in experimental studies for testing antiepileptic drugs, but further characterization of this model is essential for its usage in testing novel drugs. The aim of our study was to study the behavioral and EEG characteristics of acute seizures caused by different doses of pilocarpine in rats. *Materials and Methods*: Male Wistar rats were treated with a single intraperitoneal dose of 100 mg/kg (P100), 200 mg/kg (P200), or 300 mg/kg (P300) of pilocarpine, and epileptiform behavior and EEG changes followed within 4 h. *Results*: The intensity and the duration of seizures were significantly higher in P300 vs. the P200 and P100 groups, with status epilepticus dominating in P300 and self-limiting tonic–clonic seizures in the P200 group. The seizure grade was significantly higher in P200 vs. the P100 group only during the first hour after pilocarpine application. The latency of seizures was significantly shorter in P300 and P200 compared with P100 group. *Conclusions*: Pilocarpine (200 mg/kg) can be used as a suitable model for the initial screening of potential anti-seizure medications, while at a dose of 300 mg/kg, it can be used for study of the mechanisms of epileptogenesis.

## 1. Introduction

Epilepsy is a disorder of the central nervous system characterized by spontaneous recurrent seizures, which occur as a result of sudden, abnormal electrical activity in certain areas of the cerebral cortex [1,2]. Although the presence of seizures is the main feature of epilepsy, this disorder includes other changes at different levels of the central nervous system. Cognitive disorders, behavioral changes, and mood disorders are also common in people with epilepsy [2].

The processes of epileptogenesis and ictogenesis both play an important role in the development of seizures. Epileptogenesis is a chronic process of neuronal network remodeling at the structural, cellular, and molecular levels, which increases the susceptibility of neurons to produce spontaneous electrical discharges and may be caused by genetic or acquired factors [3,4]. The exact mechanisms of epileptogenesis have not yet been sufficiently clarified, but based on the model of temporal lobe epilepsy (TLE), it has been shown that neurodegeneration with reactive gliosis, axonal sprouting with the formation of dysfunctional synaptic connections, neuroinflammation, neurogenesis, glial dysfunction, and extracellular matrix changes are all involved in this process [3]. Ictogenesis, on the other hand, describes the mechanisms of individual seizure generation and the events leading to the transition from the interictal to ictal period. These processes may be diverse in various types of seizures, and involved mechanisms include disturbed GABAergic and glutamatergic transmission, ionic perturbations, altered function and/or structure of pyramidal cells, interneurons, and astrocytes [5]. 

Although the mechanisms of epileptogenesis are partly known, there are no available therapeutic procedures that can slow the process of epileptogenesis. Therapy for epilepsy is mainly focused on the process of ictogenesis, which refers to the prevention of discharge in already remodeled brain structures. Therefore, there is a need to establish new experimental models that could explain the mechanisms of epileptogenesis but can also provide a basis for the study of novel anti-seizure medications. Most of the currently used models study the mechanisms of ictogenesis without epileptogenesis [6]. These models include homocysteine-induced epilepsy [7], the pentylenetetrazole model [8], the metaphit-induced model [9], penicillin-induced epilepsy [10], and the chemical and electrophysiological kindling models [11,12]. These models mainly cause an imbalance between excitatory and inhibitory transmission [13]. Although numerous anti-seizure medications have been tested in different experimental models, approximately 30% of patients have epilepsy refractory to all drugs, indicating that modern antiepileptic therapy has limited efficacy [14,15]. Focal seizures occurring in temporal lobe epilepsy are among the most intractable types of seizures for currently available therapeutic modalities [16,17]. This is the reason why the most widely used models for the testing of antiepileptic drugs are temporal lobe epilepsy models [17]. Additionally, it is widely known that some patients with the same type of seizures respond to the therapy, while others do not. For this reason, the epilepsy models may be divided into the models used for the identification of responders and non-responders to the therapy and the models that are per se resistant to some anti-seizure medications [17]. Among the models used for the differentiation between responders and non-responders is pilocarpine-induced model. Another model used for the same purpose is the amygdala kindling model [17].

The pilocarpine model, introduced in the early 1980s, is a well-established model in epilepsy research due to its ability to reliably induce status epilepticus (SE) in rodents, closely mimicking human temporal lobe epilepsy [18]. The acute administration of pilocarpine triggers a sequence of behavioral changes that begins with gustatory and olfactory automatisms and progresses to motor limbic seizures [18]. Within 2 h, pilocarpine induces SE, which initiates neural network remodeling and epileptogenesis—a process linked with neurodegeneration and gliosis in regions such as the hippocampus, thalamus, olfactory cortex, amygdala, neocortex, and substantia nigra [18,19]. SE is initially induced by the activation of muscarinic M1 receptors and maintained through NMDA receptor activation [18].

It has been confirmed that the pilocarpine model in rats and mice may be used for the identification of responders and non-responders to levetiracetam [20] and phenobarbital [21]. However, the optimal dose of pilocarpine for testing the response to drugs has not been clearly established. While initial studies revealed that 400 mg/kg of pilocarpine caused SE with epileptogenesis [18], the major shortcoming of this model was high mortality. For this reason, Glien et al. (2001) introduced a repeated-use model with an initial dose of 200 mg/kg and subsequent repeated doses of 100 mg/kg until the development of SE [22]. However, this modification did not reduce mortality and is also not efficient in all animal models. Administration of 100 mg/kg to mice up to 800 mg/kg did not consistently induce SE and epileptogenesis [23]. The high mortality rate and high variability in SE induction have led to the development of the lithium–pilocarpine model, consisting of a single pretreatment with lithium and the repeated use of low doses of pilocarpine (10 mg/kg) every 30 min [22]. Although this model has shown improved consistency in SE induction and low mortality, the lithium–pilocarpine model is rather time consuming, and there are still wide inter-strain variations in SE development [24,25]. Even within the same strain, results have been different from different breeding laboratories [26,27]. For this reason, the pilocarpine model still remains popular even in the new era of epilepsy research and requires further specification [28]. Recently, a novel screening program for testing new antiepileptics (The New Epilepsy Therapy Screening Program, ETSP) was developed, consisting of two phases: an Identification phase and a Differentiation phase [29]. The possible usage of the pilocarpine model in this program has still not been established. Additionally, the usage of this model in the study of ictogenesis has not been sufficiently clarified. 

Based on previous research, the aim of the present study was to investigate the behavioral and EEG characteristics of acute seizures caused by different doses of pilocarpine in rats and to test the possible translational potential of this model in the research of novel anti-seizure medications and mechanisms of ictogenesis and epileptogenesis. 

## 2. Materials and Methods

Experiments were performed on adult male Wistar rats, 6–8 weeks old, weighing 170–200 g, bred at the Military Medical Academy Breeding Laboratories, Belgrade, Serbia. The animals were placed in transparent plastic cages (55 × 35 × 30 cm; 2 animals per cage) and kept in standard laboratory conditions (temperature 22 ± 2 °C, relative humidity 50%, 12/12 h light/dark cycles, with the light turning on at 9 a.m.), with free access to standard laboratory animal chow and tap water. Before the experiment, animals were placed in separate cages for better monitoring of behavioral and EEG changes. All experimental procedures were carried out in accordance with the European Council Directive (2010/63/EU) and approved by The Ethical Committee of the University of Belgrade, Belgrade, Serbia (Permission No 1891/2). 

In order to examine the effect of pilocarpine on the behavioral and EEG characteristics of acute epileptic seizures, all of the animals (n = 24) were randomly divided into three groups (n = 8 per group), which received different doses of pilocarpine: 100 mg/kg (P100), 200 mg/kg (P200), and 300 mg/kg (P300). Before administration, pilocarpine (Sigma Aldrich, Co., Darmstadt, Germany) was dissolved in saline (0.9% NaCl) and administered intraperitoneally at a volume of 0.1 mL/100g of body weight [22]. Pilocarpine was injected at 9 a.m. Behavioral changes were monitored for 4 h after pilocarpine administration and included the incidence, latency, and intensity of the seizures. The latency of seizure is defined as the time from pilocarpine administration to the first seizure response. The severity of seizure was graded by the modified descriptive rating scale according to Racine (1972) [30] and was expressed using a grade from 0 to 4: 0—no seizures; 1—head nodding, lower jaw twitching; 2—myoclonic body jerks (hot plate reaction) and bilateral forelimb clonus with full rearing (Kangaroo position); 3—progression to generalized clonic convulsions followed by tonic extension of fore and hind limbs and tail; 4—status epilepticus, which implies a prolonged generalized tonic–clonic seizure lasting longer than 15 s or a series of repeated clonic seizures lasting longer than 5 min [31]. The severity of grade was assessed over 4 h, and maximal severity was analyzed in 1 h intervals.

### 2.1. EEG Recordings

For EEG recordings, rats were anesthetized with ketamine (100 mg/kg i.p.) dissolved in saline (0.9% NaCl), and three gold-plated electrodes were implanted over the frontal (LF: 2 mm in front of the bregma and 2 mm left to the middle line), parietal (RP: 2 mm in front of the lambda and 2 mm right to the middle line), and occipital cortices (2 mm behind lambda) using the stereotaxic method. Animals were left for 7 days to recover after the implantation of electrodes in order to avoid interference between the effects of surgery and pilocarpine on EEG activity, and they were treated locally with vancomycin powder to prevent infection. An 8-channel EEG apparatus (RIZ, Zagreb, Croatia) was used for the registration. The signals were digitized using a SCB-68 data acquisition card (National Instruments Co., Austin, TX, USA). A sampling frequency of 512 Hz/channel and 16-bit analogous-to-digital (A/D) conversion were used for the EEG signals. The cutoff frequencies for EEG recordings were set at 0.3 and 100 Hz for the high-pass and low-pass filters, respectively. Ambient noise was eliminated using a 50 Hz notch filter. Data acquisition and signal processing were performed on LabVIEW based software which was previously developed in our laboratory (NeurosciLaBG-EEG v.3.0) [32]. After acquisition, the number and duration of pilocarpine-induced seizures per hour was determined consistently in LF-RP lead.

### 2.2. Statistical Analysis

The incidence of seizures was expressed as a percentage, while the latencies and seizure grades were expressed as medians with the 25th and 75th percentiles in parentheses. Since the normal distribution of latencies, number, and duration of seizures per hour was not established by the Kolmogorov–Smirnov test, the significance of the difference was estimated by the nonparametric tests. The difference in severity grades, number, and duration of seizures at different time points was analyzed by the Friedman test with the Dunn–Bonferroni post-hoc test. The difference in latencies was estimated by the Kruskall–Wallis test with the Mann–Whitney post-hoc test. Fisher’s exact probability test was used to assess the significance of the difference in seizure incidence. Statistical analysis was performed using GraphPad Prism 6.0, and the difference was considered statistically significant if *p* < 0.05.

## 3. Results

### 3.1. Behavioral Analysis

**Figure 1:**
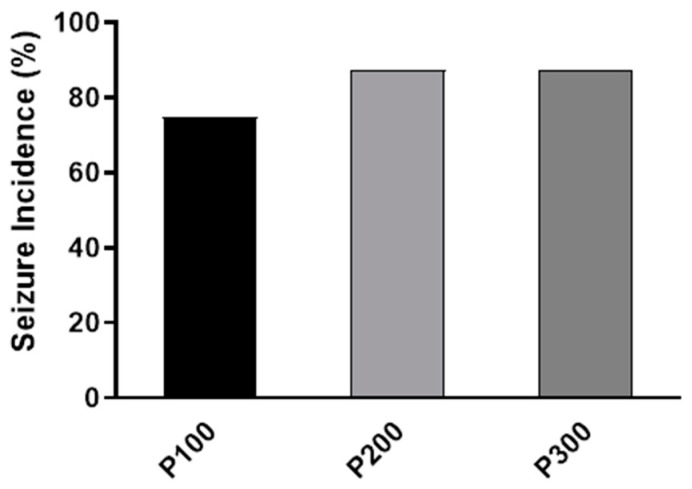
The incidence of seizures (%) caused by a single intraperitoneal administration of pilocarpine at a dose of 100 mg/kg (P100), 200 mg/kg (P200), or 300 mg/kg (P300). The exact probability test was used to assess the significance of the difference (*p* = 0.6458).

No abnormal behavior was evident in animals before pilocarpine treatment. Convulsive seizures were observed in all pilocarpine-treated groups within 4 h after pilocarpine administration. The highest incidence of seizures was found in the P300 and P200 groups (7/8, 87.5%), while in the P100 group, the seizure incidence was 6/8 (75%). However, the seizure incidence was not significantly different between pilocarpine-treated groups (*p* = 0.7408; Figure 1). 

**Figure 2:**
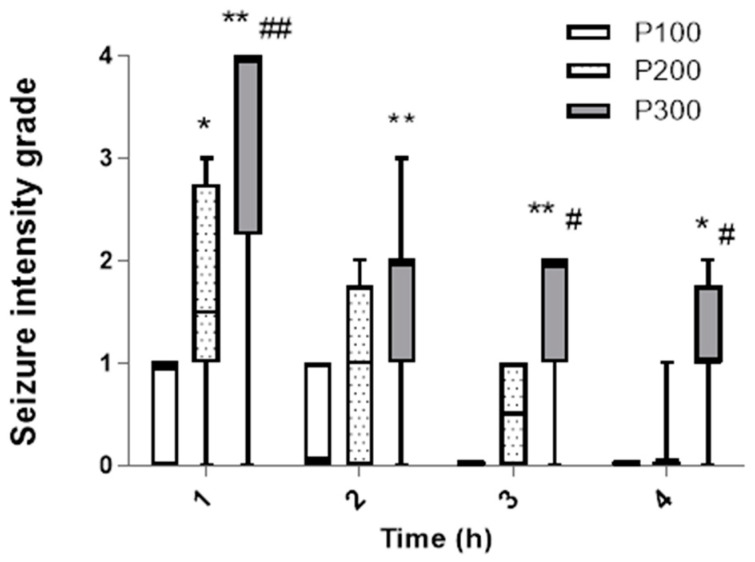
The intensity of seizures induced by a single administration of pilocarpine in groups P100, P200, and P300 within 4 h after treatment. The intensity was assessed according to the descriptive scale and expressed as a grade from 0 to 4: 0—no seizures; 1—head nodding and clonic jaw twitching; 2—myoclonic body jerks (hot plate reaction), bilateral forelimb clonus with full rearing (Kangaroo position); 3—progression to generalized clonic convulsions followed by tonic extension of fore and hind limbs and tail; 4—prolonged severe tonic–clonic convulsions lasting over 15 s (status epilepticus) or frequent repeated episodes of clonic convulsions for an extended period of time (over 5 min). The significance of the difference was estimated by Friedman with Dunn–Bonferroni post-hoc test (* *p* < 0.05 vs. P100, # *p* < 0.05 vs. P200, ** *p* < 0.001 vs. P100, ## *p* < 0.001 vs. P200). For detailed information, see Figure 1.

In contrast to the incidence, the seizure intensity during the first hour was significantly higher in the group treated with pilocarpine at a dose of 300 mg/kg [4 (2, 3.75)] in comparison with the groups that received 200 mg/kg [1.5 (1, 2.75)] (*p* = 0.0005) and 100 mg/kg [1 (0, 1)] (*p* < 0.0001, Figure 2). The dominant seizure grade in the P300 group was 4 (five animals developed SE), and in P200, grades 2 and 1 were dominant, while in the P100 group, only grade 1 was observed. Also, during the first hour, the seizure intensity was significantly higher in P200 compared with the P100 group (*p* = 0.048). Over time, the seizure intensity decreased in all groups. However, during the second, third, and fourth hour, the seizure intensity was still significantly higher in P300 in comparison with the P100 group. In the third and fourth hour, the median seizure grade was also significantly higher in P300 compared with the P200 group (*p* < 0.05). After the first hour, there was no significant difference in the seizure intensity between the groups that received 200 mg/kg and 100 mg/kg of pilocarpine (*p* > 0.05). In the third and fourth hour, animals did not develop seizures in the P100 group, while in the P200 group, the highest seizure grade was 1 (Figure 2). In the P100 and P200 groups, no SE was evident after pilocarpine administration, and the seizures ceased within a 4 h interval in these groups. 

**Figure 3:**
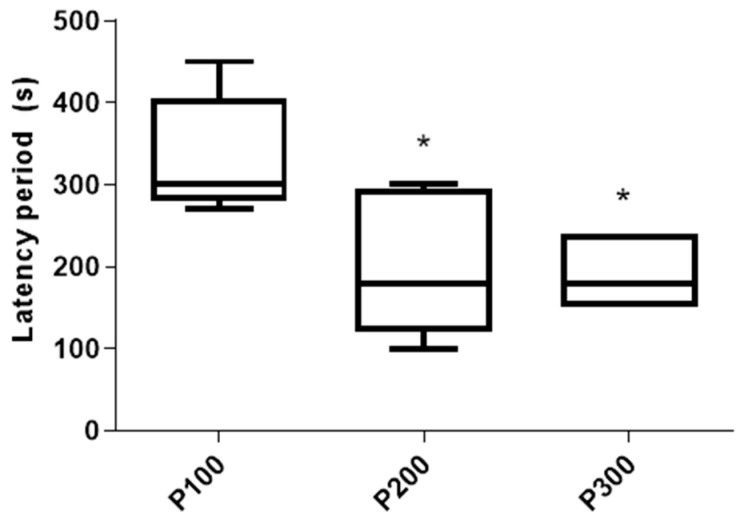
Latency period (s) of seizures caused by a single administration of pilocarpine in the P100, P200, and P300 groups. Latency period is defined as a time between pilocarpine administration and the first seizure sign. The significance of the difference was estimated by Kruskall–Wallis with Mann–Whitney post-hoc test (* *p* < 0.05 vs. P100). For detailed information, see Figure 1.

The latency of the seizures was significantly shorter in P300 [180 s (150 s, 240 s)] (*p* = 0.022) and P200 [180 s (120 s, 295 s)] (*p* = 0.0498) in comparison with the P100 group [300 s (280 s, 405 s)]. On the other hand, the latency of the seizures was not significantly different between the P300 and P200 groups (*p* > 0.999; Figure 3). The mortality rate in the P300 group was 3/8 (37.5%), and no mortality was evident in the P200 or P100 groups 4 h after pilocarpine administration.

### 3.2. EEG Analysis

EEG analysis showed that the average duration of the seizures was significantly longer in P300 in comparison with the P100 (*p* < 0.0001) and P200 groups (*p* < 0.0001) within the first hour after pilocarpine administration. In the second hour, no significant difference in the seizure duration was found among groups (*p* > 0.05). However, in the third and fourth hour, the average seizure duration was significantly longer in P300 when compared with the P100 group. On the other hand, there were no differences in seizure duration between P300 and P200 (*p* > 0.05) or between the P200 and P100 groups (*p* > 0.05) in the third or fourth hour after pilocarpine administration (Table 1). 

Although the number of seizures progressively increased with increasing doses of pilocarpine, no significant difference in seizure number was evident between groups within 4 h after treatment (*p* > 0.05; Table 2).

**Figure 4:**
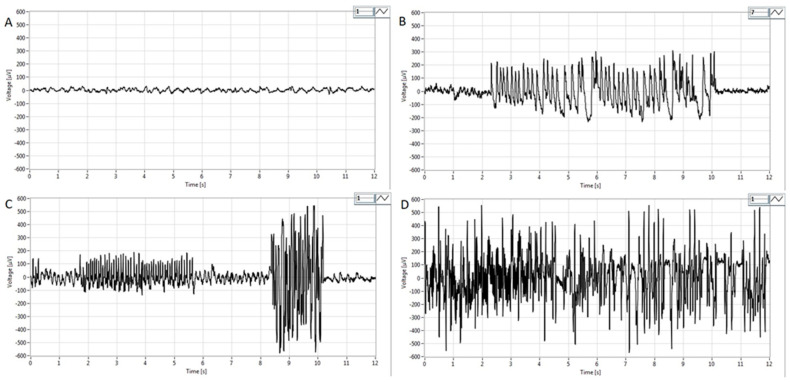
Representative EEG findings in pilocarpine-treated groups obtained in the first hour after pilocarpine treatment from the parietal cortex. (**A**) Note activity without signs of epileptiform discharges. Basal EEG activity in the beta band (14–30 Hz) corresponding to the physiological behavior before pilocarpine administration. (**B**) Intermittent paroxysmal polyspikes and spike–wave complexes in the P100 group. (**C**) Epileptiform spike bursts of variable amplitude lasting from 2.2 to 14.1s in the P200 group and high voltage spikes with amplitudes of more than 300–500 μV. (**D**) High-voltage, high-frequency polyspike burst activity combined with intermittent spike–wave complexes lasting for up to 200 s, corresponding to status epilepticus. For detailed information, see Figure 1.

Representative EEG tracings are presented in Figure 4. Before pilocarpine administration, low-voltage, high-frequency activity in the beta range (14–30 Hz) without epileptiform discharges was evident on the EEG (Figure 4A), corresponding to an awake state with psychophysical activity. However, in the P100 group during the first hour after the treatment, paroxysmal spikes and spike–wave complexes appeared on the EEG (Figure 4B). In the P200 group, polyspikes with total durations between 2.2 s and 14.1 s were observed (Figure 4C). In contrast to the P100 and P200 groups, i.p. administration of pilocarpine (300 mg/kg) caused high-voltage, high-frequency polyspikes in combination with spike–wave discharges lasting up to 200 s, corresponding to generalized tonic–clonic SE (Figure 4D).

## 4. Discussion

The results of our study show that the pilocarpine-induced model of epilepsy in rats can potentially be used to study the mechanisms of both ictogenesis and epileptogenesis. Although the seizure incidence and the number of seizures did not differ significantly between groups treated with various doses of pilocarpine (Figure 1, Table 2), the seizure intensity, latency, and duration were affected in a dose-dependent manner (Figure 2 and Figure 3, Table 1). The dose of 300 mg/kg consistently induced SE, indicating that this dose may be used for the study of epileptogenesis, since it is well known that SE initiates epileptogenesis in pilocarpine-treated rats [18]. Additionally, the consistent appearance of SE qualifies this dose for the identification of responders and non-responders in the testing of potential new anti-seizure medications [17]. On the other hand, pilocarpine (200 mg/kg) cannot be used for the research of epileptogenesis mechanisms since this dose caused no SE in this group (Figure 2). However, 200 mg/kg consistently induced seizures of lower intensities, indicating that this dose may be used for the study of ictogenesis mechanisms, as well as in the initial screening of potential new anti-seizure medications. 

The utility of the pilocarpine model in research on the mechanisms of epileptogenesis has been widely recognized. Ahmed et al. (2021) used pilocarpine to induce SE in male FVB/NJ mice with an initial dose of 200 mg/kg, followed by additional doses of 100 mg/kg if SE was not induced within 1 h [28]. The animals were then monitored at various time points up to two weeks post-SE to assess the progression of epileptogenesis. Similarly, in a study by Luna-Munguia et al. (2019), pilocarpine was administered to induce SE at a dose of 340 mg/kg, followed by an additional 170 mg/kg if SE was not induced within 40 min [33]. The animals were then observed for 14 days post-SE to evaluate the development of spontaneous seizures and significant biochemical changes. These studies underscore the effectiveness of the pilocarpine model in both inducing SE and providing insights into the molecular mechanisms underlying epileptogenesis. Our results are in accordance with previous studies, and we can recommend pilocarpine (300 mg/kg) for studying the mechanisms of epileptogenesis.

However, this is the first study to our knowledge that suggested the use of pilocarpine (200 mg/kg) for the study of the mechanistic aspects of ictogenesis. The prerequisite for the usage of the model in studying ictogenesis is the consistent appearance of seizures with reproducible behavioral and EEG features. Currently, pentylenetetrazol (PTZ), strychnine, and homocysteine are used for this purpose. Seizures provoked by pilocarpine (200 mg/kg) also share some similarities with those induced by PTZ, strychnine, and homocysteine, although the underlying mechanisms differ. While PTZ inhibits GABA_A_ receptors, pilocarpine stimulates muscarinic and NMDA glutamatergic receptors [34]. Recent studies have suggested that the imbalance of AMPA receptor phosphorylation and signaling pathways in specific regions of the hippocampus following pilocarpine administration may be crucial in the development of seizures. The dorsal hippocampus in particular may be more vulnerable to impaired glutamate uptake and gliosis, which could be significant for identifying seizure onset sites and establishing therapeutic targets for epilepsy treatment [35]. Therefore, pilocarpine-induced seizures (200 mg/kg) could serve as an optional model for further research on ictogenesis mechanisms, since it is still not clear whether preictal-to-ictal transition follows the same pathway in all seizures, and the mechanisms of pilocarpine- and PTZ-induced seizures may not be identical. Additionally, the grading scale used for homocysteine-induced seizures could also be applied to pilocarpine-induced seizures, as the mechanisms of action in epileptogenesis and ictogenesis for both agents are similar [31]. Nonetheless, further research is needed for a more accurate comparison of these models.

Pilocarpine (200 mg/kg) may also be used for the initial screening of potential novel anti-seizure medications. Screening of potential anti-seizure medications is performed in acute seizure models using a structurally and functionally intact brain [29,36]. For this purpose, maximal electroshock seizure (MES) test and 6 Hz test may be used [34]. In the MES model, there is an increase in the expression of the Homer1a gene, which regulates the level of metabotropic glutamate receptors, suggesting that MES and pilocarpine may share some similar mechanisms of seizure generation [37]. However, seizures induced by pilocarpine (200 mg/kg) tend to have lower severity compared to MES seizures and are primarily characterized by bilateral clonic spasms of the forelimbs with rearing to the hind limbs, with only the occasional appearance of generalized tonic–clonic seizures [37]. These convulsions were associated with paroxysms of polyspike activity of variable amplitudes on EEG, and importantly, no lethal outcomes were observed after the administration of 200 mg/kg pilocarpine.

One promising approach is to incorporate the pilocarpine model as an alternative epilepsy model in ETSP. This program includes the Identification phase for the screening of potential novel anti-seizure medications and the Differentiation phase, which enables the detailed study of the effects of potential anti-seizure medications on various clinical features of epilepsy [38]. In the Differentiation phase, chronic epilepsy models are used that are resistant to some anti-seizure drugs. Currently used models include the intrahippocampal kainate model of mesial TLE in mice, the lamotrigine-resistant amygdala kindled rat, and a chronic epilepsy rat model in which epilepsy develops after the induction of SE by a single dose of kainate [39]. The benzodiazepine-resistant lithium–pilocarpine SE model in rats has also been proposed as an ancillary pharmacoresistant model for testing in the Differentiation phase [17]. Interestingly, few studies have investigated the pharmacoresistance of spontaneous recurrent seizures (SRS) in pilocarpine models. Leite and Cavalheiro (1995) found that pilocarpine-induced SRS are resistant to ethosuximide but are not resistant to carbamazepine, phenytoin, phenobarbital, or valproate [40]. However, the tolerance to carbamazepine, valproate, and phenobarbital has been observed in the second week of treatment. Similarly, Chakir et al. (2006) [41] found that SRS caused by pilocarpine can be effectively blocked with the prompt initiation of carbamazepine therapy, but the resistance develops if carbamazepine administration is delayed. Therefore, there is a basis for the usage of pilocarpine-induced epilepsy in the Differentiation phase of ETSP. 

The present study, however, does not add new information regarding the potential usage of pilocarpine model in the Differentiation phase. Behavioral and EEG analysis showed that pilocarpine at a dose of 300 mg/kg causes predominantly generalized tonic–clonic SE similar to kainate, which is used in ETSP [42]. Additionally, pilocarpine and kainate induce similar morphological changes in the olfactory cortex, amygdala, thalamus, neocortex, hippocampus, and substantia nigra [43,44]. One advantage of pilocarpine (300 mg/kg) over the kainate model is the shorter latency period between the SE and the development of the first spontaneous seizure [45]. The intrahippocampal kainate model of TLE is also suitable for the testing of anti-seizure medications because it is resistant to many previous antiepileptics and because it causes very frequent epileptiform changes in the EEG that do not require long-term monitoring [46,47,48]. However, the pilocarpine model could ensure more consistent results than the kainate TLE model, since the variability of seizure manifestations is lower in the pilocarpine model [13]. According to the similarity between these models and the results of the present study, we propose pilocarpine (300 mg/kg) for further testing of pharmacoresistance to new anti-seizure drugs to provide novel information on whether this dose has the potential to be used in the Differentiation phase of ETSP.

Pilocarpine at a dose of 100 mg/kg caused only occasional, short-term clonic and myoclonic lower jaw muscle spasms (grade 1) during the first two hours after administration. Due to such behavioral changes, this dose cannot be used to investigate the therapeutic effects of the potential anti-seizure medications and neuroprotective substances. However, occasional spikes and spike–wave complexes were evident after the injection of 100 mg/kg pilocarpine, making this dose adequate for the study of ictogenesis. Microelectrode registration of the brain electrical activity revealed that epileptiform discharges may be detected in limited parts of the brain even before clinical manifestations of seizures (microseizures), and they can be involved in the transition from the interictal to the ictal period. The period when microseizures are generated is known as the preictal period, and information on the foci involved in the initiation of microseizures, as well as on the molecular mechanisms of their generation, would supplement current knowledge on ictogenesis [49]. Although pilocarpine (100 mg/kg) cannot be used for the testing of anticonvulsive substances, this model may be used for further clarification of the cellular, molecular, and electrophysiological events in the preictal period.

One of the limitations of the pilocarpine-induced model is the inability to reliably extrapolate the obtained results to the human population, as the most common causes of epilepsy (brain trauma, cerebrovascular disease, tumors, and CNS inflammation) trigger different mechanisms of epileptogenesis than those induced by pilocarpine. Consequently, new epilepsy models are being developed, including models caused by stroke, traumatic brain injury, and viral encephalitis in mice [50,51,52,53]. However, these models are still in the early stages of development and require further validation before they can be widely adopted. An additional limitation of our study that must be acknowledged is a small sample size and the short 4 h follow-up period. These factors may impact the generalization of our findings, emphasizing the need for future studies to include larger sample sizes and extended observation periods to better understand the long-term effects of pilocarpine-induced seizures and their relevance to human epilepsy.

## 5. Conclusions

Based on the results of our study, it can be concluded that pilocarpine induces seizures in a dose-dependent manner. Pilocarpine at a dose of 100 mg/kg may be used for the further study of the ictogenesis mechanisms, especially early events in the preictal period, but cannot be used for drug testing. On the other hand, 200 mg/kg can be used as a suitable model for the initial testing of potential anti-seizure medications, primarily those that may inhibit the occurrence of generalized tonic–clonic seizures. Additionally, this dose may also be used for the investigation of ictogenesis. Finally, at a dose of 300 mg/kg, pilocarpine predominantly causes the progression of seizures to SE and can be used for studying the mechanisms of epileptogenesis.

## Figures and Tables

**Table 1:** The effect of pilocarpine dose on seizure duration. Pilocarpine in doses of 100 mg/kg (P100), 200 mg/kg (P200), and 300 mg/kg (P300) was administered intraperitoneally, and seizure duration was determined by EEG Software (NeusrosciLaBG-EEG v 3.0) over 4 h.

Time (h)	Seizure Duration (s)		
	P100	P200	P300
1	2.5 (2.1, 3.4)	4.9 (2.3, 10.5)	30.4 (20.1, 70.25) **#
2	2.6 (1.9, 3.5)	10.05 (7.6, 11.75)	9.35 (5.975, 11.65)
3	0 (0, 0)	2.2 (1.6, 4.2)	8 (5, 9.95) **
4	0 (0, 0)	1.6 (1.2, 2.55)	4.2 (2.725, 6.175) *

The results are presented as medians with 25th and 75th percentiles in parentheses. The significance of the difference was estimated by Friedman test with Dunn–Bonferroni post-hoc test (* *p* < 0.05, ** *p* < 0.01 vs. P100, # *p* < 0.05 vs. P200).

**Table 2:** The effect of pilocarpine dose on the number of seizures. Pilocarpine in doses of 100 mg/kg (P100), 200 mg/kg (P200), and 300 mg/kg (P300) was administered intraperitoneally, and the number of seizures was determined by EEG Software (NeuroSciLab) over 4 h.

Time (h)	The Number of Seizures (n)		
	P100	P200	P300
1	9 (1.75, 12.5)	22.5 (17.75, 28)	26.5 (18, 30.25)
2	0 (0, 3.75)	8.5 (0, 10.75)	20 (17.25, 22.75)
3	0 (0, 0)	2.5 (0, 5.75)	9.5 (4.75, 13.25)
4	0 (0, 0)	0 (0, 1.5)	8.5 (6.5, 10)

The results are presented as medians with 25th and 75th percentiles in parentheses. No significant difference between groups was found.

## Data Availability

The original contributions presented in the study are included in the article, further inquiries can be directed to the corresponding authors.

## References

[1] Thodeson D.M., Brulet R., Hsieh J. (2018). Neural stem cells and epilepsy: Functional roles and disease-in-a-dish models. Cell Tissue Res..

[2] Minjarez B., Camarena H.O., Haramati J., Rodríguez-Yañez Y., Mena-Munguía S., Buriticá J., García-Leal O. (2017). Behavioral changes in models of chemoconvulsant-induced epilepsy: A review. Neurosci. Biobehav. Rev..

[3] Sharma S., Puttachary S., Thippeswamy T. (2019). Glial source of nitric oxide in epileptogenesis: A target for disease modification in epilepsy. J. Neurosci. Res..

[4] Pitkänen A., Lukasiuk K., Dudek F.E., Staley K.J. (2015). Epileptogenesis. Cold Spring Harb. Perspect. Med..

[5] Blauwblomme T., Jiruska P., Huberfeld G. (2014). Mechanisms of ictogenesis. Int. Rev. Neurobiol..

[6] Aronica E., Bauer S., Bozzi Y., Caleo M., Dingledine R., Gorter J.A., Henshall D.C., Kaufer D., Koh S., Löscher W. (2017). Neuroinflammatory targets and treatments for epilepsy validated in experimental models. Epilepsia.

[7] Hrncic D., Rasic-Markovic A., Macut D., Mladenovic D., Susic V., Djuric D., Stanojlovic O. (2018). Sulfur-Containing Amino Acids in Seizures: Current State of the Art. Curr. Med. Chem..

[8] Löscher W. (2017). The Search for New Screening Models of Pharmacoresistant Epilepsy: Is Induction of Acute Seizures in Epileptic Rodents a Suitable Approach?. Neurochem. Res..

[9] Stanojlović O.P., Zivanović D.P., Mirković S.D., Mikhaleva I.I. (2005). Antiepileptic activity of delta sleep-inducing peptide and its analogue in metaphit-provoked seizures in rats. Seizure.

[10] De Deyn P.P., D’Hooge R., Marescau B., Pei Y.Q. (1992). Chemical models of epilepsy with some reference to their applicability in the development of anticonvulsants. Epilepsy Res..

[11] Samokhina E., Samokhin A. (2018). Neuropathological profile of the pentylenetetrazol (PTZ) kindling model. Int. J. Neurosci..

[12] Gorter J.A., van Vliet E.A., Lopes da Silva F.H. (2016). Which insights have we gained from the kindling and post-status epilepticus models?. J. Neurosci. Methods.

[13] Lévesque M., Avoli M. (2013). The kainic acid model of temporal lobe epilepsy. Neurosci. Biobehav. Rev..

[14] Radu B.M., Epureanu F.B., Radu M., Fabene P.F., Bertini G. (2017). Nonsteroidal anti-inflammatory drugs in clinical and experimental epilepsy. Epilepsy Res..

[15] Scorza F.A., Arida R.M., Naffah-Mazzacoratti M.G., Scerni D.A., Calderazzo L., Cavalheiro E.A. (2009). The pilocarpine model of epilepsy: What have we learned?. An. Acad. Bras. Cienc..

[16] Schmidt D., Löscher W. (2005). Drug Resistance in Epilepsy: Putative Neurobiologic and Clinical Mechanisms. Epilepsia.

[17] Löscher W., White H.S. (2023). Animal Models of Drug-Resistant Epilepsy as Tools for Deciphering the Cellular and Molecular Mechanisms of Pharmacoresistance and Discovering More Effective Treatments. Cells.

[18] Turski W.A., Cavalheiro E.A., Schwarz M., Czuczwar S.J., Kleinrok Z., Turski L. (1983). Limbic seizures produced by pilocarpine in rats: Behavioural, electroencephalographic and neuropathological study. Behav. Brain Res..

[19] Nirwan N., Vyas P., Vohora D. (2018). Animal models of status epilepticus and temporal lobe epilepsy: A narrative review. Rev. Neurosci..

[20] Glien M., Brandt C., Potschka H., Löscher W. (2002). Effects of the novel antiepileptic drug levetiracetam on sponta-neous recurrent seizures in the rat pilocarpine model of temporal lobe epilepsy. Epilepsia.

[21] Bankstahl M., Bankstahl J.P., Löscher W. (2012). Inter-individual variation in the anticonvulsant effect of phenobarbital in the pilocarpine rat model of temporal lobe epilepsy. Exp. Neurol..

[22] Glien M., Brandt C., Potschka H., Voigt H., Ebert U., Löscher W. (2001). Repeated low-dose treatment of rats with pilocarpine: Low mortality but high proportion of rats developing epilepsy. Epilepsy Res..

[23] Neumann A., Abele J., Kirschstein T., Engelmann R., Sellmann T., Köhling R., Müller-Hilke B. (2017). Mycophenolate mofetil prevents the delayed T cell response after pilocarpine-induced status epilepticus in mice. PLoS ONE.

[24] Winawer M.R., Makarenko N., McCloskey D.P., Hintz T.M., Nair N., Palmer A.A., Scharfman H.E. (2007). Acute and chronic responses to the convulsant pilocarpine in DBA/2J and A/J mice. Neuroscience.

[25] Chen J., Larionov S., Pitsch J., Hoerold N., Ullmann C., Elger C.E., Schramm J., Becker A.J. (2005). Expression analysis of metabotropic glutamate receptors I and III in mouse strains with different susceptibility to experimental temporal lobe epilepsy. Neurosci. Lett..

[26] Bankstahl M., Brandt C., Löscher W. (2009). Differences in pilocarpine-induced epileptogenesis and behavioral alterations of Sprague-Dawley rats from two different breeders. Naunyn-Schmiedeberg’s Arch. Pharmacol..

[27] Schauwecker P.E. (2012). Strain differences in seizure-induced cell death following pilocarpine-induced status epilepticus. Neurobiol. Dis..

[28] Ahmed M.M., Carrel A.J., Cruz Del Angel Y., Carlsen J., Thomas A.X., González M.I., Gardiner K.J., Brooks-Kayal A. (2021). Altered Protein Profiles During Epileptogenesis in the Pilocarpine Mouse Model of Temporal Lobe Epilepsy. Front. Neurol..

[29] Löscher W. (2017). Animal Models of Seizures and Epilepsy: Past Present and Future Role for the Discovery of Antiseizure Drugs. Neurochem. Res..

[30] Racine R.J. (1972). Modification of seizure activity by electrical stimulation: II. Motor seizure. Electroencephalogr. Clin. Neurophysiol..

[31] Stanojlović O., Rasić-Marković A., Hrncić D., Susić V., Macut D., Radosavljević T., Djuric D. (2009). Two types of seizures in homocysteinethiolactone-treated adult rats behavioral and electroencephalographic study. Cell Mol. Neurobiol..

[32] Mladenovic D., Hrncic D., Rasic-Markovic A., Puskas N., Petrovich S., Stanojlovic O. (2013). Spectral analysis of thioacetamide-induced electroencephalographic changes in rats. Hum. Exp. Toxicol..

[33] Luna-Munguia H., Zestos A.G., Gliske S.V., Kennedy R.T., Stacey W.C. (2019). Chemical biomarkers of epileptogenesis and ictogenesis in experimental epilepsy. Neurobiol. Dis..

[34] Meral I., Esrefoglu M., Dar K.A., Ustunova S., Aydin M.S., Demirtas M., Arifoglu Y. (2016). Effects of Nigella sativa on apoptosis and GABAA receptor density in cerebral cortical and hippocampal neurons in pentylenetetrazol induced kindling in rats. Biotech. Histochem..

[35] Lopes M.W., Lopes S.C., Costa A.P., Gonçalves F.M., Rieger D.K., Peres T.V., Eyng H., Prediger R.D., Diaz A.P., Nunes J.C. (2015). Region-specific alterations of AMPA receptor phosphorylation and signaling pathways in the pilocarpine model of epilepsy. Neurochem. Int..

[36] Löscher W. (2016). Fit for purpose application of currently existing animal models in the discovery of novel epilepsy therapies. Epilepsy Res..

[37] Cavarsan C.F., Matsuo A., Blanco M.M., Mello L.E. (2015). Maximal electroshock-induced seizures are able to induce Homer1a mRNA expression but not pentylenetetrazole-induced seizures. Epilepsy Behav..

[38] Kehne J.H., Klein B.D., Raeissi S., Sharma S. (2017). The National Institute of Neurological Disorders and Stroke (NINDS) Epilepsy Therapy Screening Program (ETSP). Neurochem. Res..

[39] Goddard G.V., McIntyre D.C., Leech C.K. (1969). A permanent change in brain function resulting from daily electrical stimulation. Exp. Neurol..

[40] Leite J.P., Cavalheiro E.A. (1995). Effects of conventional antiepileptic drugs in a model of spontaneous recurrent seizures in rats. Epilepsy Res..

[41] Chakir A., Fabene P., Ouazzani R., Bentivoglio M. (2006). Drug resistance and hippocampal damage after delayed treatment of pilocarpine-induced epilepsy in the rat. Brain Res. Bull..

[42] Hellier J.L., Patrylo P.R., Buckmaster P.S., Dudek F.E. (1998). Recurrent spontaneous motor seizures after repeated low-dose systemic treatment with kainate: Assessment of a rat model of temporal lobe epilepsy. Epilepsy Res..

[43] Turski L., Ikonomidou C., Turski W.A., Bortolotto Z.A., Cavalheiro E.A. (1989). Review: Cholinergic mechanisms and epileptogenesis. The seizures induced by pilocarpine: A novel model of intractable epilepsy. Synapse.

[44] Cavalheiro E.A., Leite J.P., Bortolotto Z.A., Turski W.A., Ikonomidou C., Turski L. (1991). Long-term effects of pilocarpine in rats: Structural damage of the brain triggers kindling and spontaneous recurrent seizures. Epilepsia.

[45] Drexel M., Preidt A.P., Sperk G. (2012). Sequel of spontaneous seizures after kainic acid-induced status epilepticus and associated neuropathological changes in the subiculum and entorhinal cortex. Neuropharmacology.

[46] Riban V., Bouilleret V., Pham-Le B.T., Fritschy J.M., Marescaux C., Depaulis A. (2002). Evolution of hippocampal epileptic activity during the development of hippocampal sclerosis in a mouse model of temporal lobe epilepsy. Neuroscience.

[47] Klein S., Bankstahl M., Löscher W. (2015). Inter-individual variation in the effect of antiepileptic drugs in the intrahippocampalkainate model of mesial temporal lobe epilepsy in mice. Neuropharmacology.

[48] Duveau V., Pouyatos B., Bressand K., Bouyssières C., Chabrol T., Roche Y., Depaulis A., Roucard C. (2016). Differential effects of antiepileptic drugs on focal seizures in the intrahippocampalkainate mouse model of mesial temporal lobe epilepsy. CNS Neurosci. Ther..

[49] Stead M., Bower M., Brinkmann B.H., Lee K., Marsh W.R., Meyer F.B., Litt B., Van Gompel J., Worrell G.A. (2010). Microseizures and the spatiotemporal scales of human partial epilepsy. Brain.

[50] Pitkänen A., Bolkvadze T., Immonen R. (2011). Anti-epileptogenesis in rodent post-traumatic epilepsy models. Neurosci. Lett..

[51] Eastman C.L., Verley D.R., Fender J.S., Temkin N.R., D’Ambrosio R. (2010). ECoG studies of valproate, carbamazepine and halothane in frontal-lobe epilepsy induced by head injury in the rat. Exp. Neurol..

[52] Eastman C.L., Verley D.R., Fender J.S., Stewart T.H., Nov E., Curia G., D’Ambrosio R. (2011). Antiepileptic and antiepileptogenic performance of carisbamate after head injury in the rat: Blind and randomized studies. J. Pharmacol. Exp. Ther..

[53] Libbey J.E., Fujinami R.S. (2011). Neurotropic viral infections leading to epilepsy: Focus on Theiler’s murine encephalomyelitis virus. Future Virol..

